# PADA-AD Test - a standardized test method for assessing takeover performance of drivers during automated driving

**DOI:** 10.1016/j.mex.2022.101901

**Published:** 2022-11-01

**Authors:** Nadja Schömig, Katharina Wiedemann, Dietrich Manstetten, Tristan Wehner, Alexandra Neukum

**Affiliations:** a Würzburg Institute for Traffic Sciences GmbH (WIVW), Robert-Bosch-Str. 4, Veitshöchheim 97209, Germany; b Robert Bosch GmbH, Renningen, Stuttgart 70465, Germany

**Keywords:** Evaluation, Human factors, HMI, Takeover performance, Automated driving, Method

## Abstract

The presented method describes a standardized test procedure for the evaluation of takeover performance of drivers during automated driving. It was primarily developed to be used for evaluating Level 3 systems (conditional automated driving). It should be applied in a driving simulator environment during the development phase of a system. The method consists of a test course on a three-lane highway with 12 test scenarios where the driver repeatedly has to take over control from the automated system. Each scenario requires the driver to build up an adequate situation awareness in order to take the decision for the correct action. The method was explicitly designed to map the four relevant steps in the takeover process of automated driving: Perception – Awareness – Decision – Action and is therefore called PADA-AD Test for automated driving. The method description contains guidelines with regard to the specification of the test course and the included test scenarios, the design and duration of the automated drives, the non-driving related task to be performed during the automated drives, the instructions to be given to the subjects and finally the measures for evaluating takeover performance of the drivers.

• A test procedure for the evaluation of takeover performance of drivers during automated driving was developed for usage in a driving simulator during the development phase of a system/HMI

• The test course enables the assessment of the driver's takeover performance in various test scenarios including higher cognitive processes
• The method is highly standardized and thus replicable through use of a predetermined test course with clearly defined scenarios, reduced environmental conditions and "popping up” of situational elements.

## Graphical abstract

**Figure:**
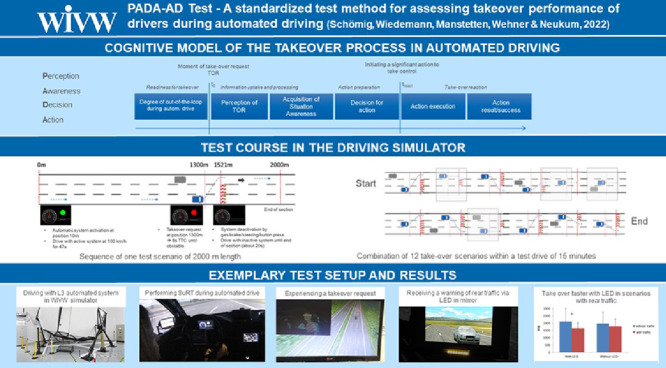

Specifications table

**Table:** 

Subject Area:	• *Engineering*
More specific subject area:	*Human factors, automated driving*
Method name:	*PADA-AD Test: A standardized test method for assessing takeover performance of drivers during automated driving*
Name and reference of original method:	*N.A.*
Resource availability:	*Wiedemann, K., Schömig, N., Wehner, T., Befelein, D. & Neukum A. (2022). Entwicklung einer standardisierten Prüfanordnung zur Bewertung der Übernahmeleistung beim automatisierten Fahren. FAT-Schriftenreihe Band 356. Available at:*https://www.vda.de/de/aktuelles/publikationen/publication/fat-schriftenreihe-356[1]

## Goals of the method

Human factor issues in automated driving continue to be a growing field of research. Despite the large number of newly published studies on various topics of the field, it is noticeable that most studies do not use a standardized procedure, for example, when investigating takeover situations. Every researcher uses their own test scenarios, test courses, side tasks and test procedures in order to assess takeover performance during automated driving. This makes it difficult to compare the results with one another. The need for developing a standardized test method to evaluate takeover performance of drivers during automated driving is therefore very high.

The method described here was derived from an analysis of the very few approaches for such standardizations that are currently published. For additional information on these approaches please read the final chapter of this paper.

The method was developed within a project funded by the Research Association of Automotive Technology (FAT; see [1] for the final report of this project with more detailed information about the method and the conducted validation studies; in German language). This consortium is part of the German Association of the Automotive Industry (VDA) and consists of all German car and commercial vehicle manufacturers and numerous suppliers in order to conduct precompetitive and joint research. The goal of the project was the development of a standardized test procedure for the evaluation of the takeover performance of drivers in automated driving. Such a method would allow to compare different HMI solutions with respect to their implication on safe driving at an early stage of product development. The intention behind the project was not re-inventing the wheel but combining best practices from past studies together into a new standardized method, which in best case will be used by all scientific institutions and OEMs in the future as standardized evaluation tool instead of the current highly individual approaches. The final chapter “Additional Information” in this paper includes a description of the already existing approaches, which have been analyzed in order to design the new method.”

In order to reach this goal, the consortium agreed on the following requirements for the method:: The consensus was to concentrate the method on evaluating systems with automation Level 3 (L3 or so called conditional automated driving; according to the classification of the Society of Automotive Engineers International SAE J3016, SAE 2018 [2]). On this automation level, the drivers are no longer required to continuously monitor the driving environment and the system's behavior. Consequently, they can take themselves “out-of-the-loop” to some extent (meaning not being in physical control of the vehicle, and not monitoring the driving situation; [3]). For example, they are allowed to direct their attention to other non-driving-related tasks or simply to relax. However, the driver still remains the fallback solution of the system in case of system failures or reaching the functional limits. In this case, the system has to issue a so-called takeover request (TOR) to the driver in order to bring him/her timely back into the loop. This takeover request usually consists of a visual and acoustic/haptic feedback via the HMI (Human Machine Interface) of the system. The regulation UN ECE R157 [4] regulates how this TOR should look like and how the system should react in case the driver does not react to that TOR (i.e. by initiating a Minimum Risk Maneuver. Furthermore, the regulation requests that L3 systems must include a function that monitors driver availability in order to detect whether they are still able to take over the driving task if necessary (see UN ECE R157 for more specifications on this driver availability monitoring system [4]).

The time budget for the takeover to be addressed by the method should be at least below 10 s (most literature on takeover reaction times in L3 assume time budgets between 3 and 7 s; see [5]). Furthermore, the method should be also able to answer Level 2 (L2) system-related research questions with regard to HMI. The main application domain of the method should be on the evaluation of system/HMI variants with regard to usability and safety issues. This implies, the method should be able to evaluate how well a driver is supported by the HMI to quickly and safely take over the driving task from the system in case of a TOR. This requires keeping the test scenario as constant as possible while manipulating influencing factors from the system or the HMI as independent variables. Variation of individual factors such as the driver state should also be possible but was not in the main focus of the method. Furthermore, the method is understood as a tool to support designers in the development phase of the system and the HMI but not for the final sign-off of the system. The method focusses on the application in the driving simulator. (Explorations on the ability to transfer the method on test tracks showed that the application will require effortful adjustments however with limited benefits for the outcomes [1]).

Additional considerations during the development phase of the method were:

• Situation awareness (i.e. the possibility to perceive, understand and anticipate the upcoming takeover situation; [6]) at the beginning of a test scenario should be kept as constant as possible across all subjects. This means that in the moment of the takeover request all subjects should be out of the loop at a comparably high level and the takeover situation should be as less predictable as possible.
• The test scenario should require a time-critical, complex takeover process by the driver within few seconds. In order to assess the performance and successful management of the situation a realistic system limit should be implemented as a plausible reason for the takeover to which the driver must react (not only merely issuing the TOR without any obvious reason in the environment).
• The optimum driver reaction to the takeover scenario should be deduced from a full interpretation of the driving scene and should not only be based on simple stimulus response patterns. Therefore, it is required to choose such scenarios or variants of scenarios which require higher cognitive resources on situation interpretation and action selection.

**Fig. 1:**
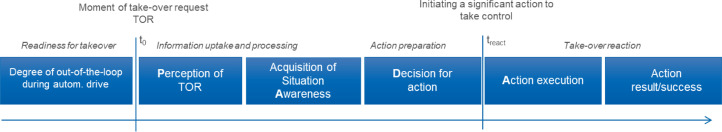
Cognitive model of the takeover process.

Based on the takeover process from [7], a model for the cognitive takeover process was developed within the project (see Fig. 1). It considers not only the timely aspects of observable takeover behavior but also the underlying cognitive processes (however, parts of the assumed processes in the graph might run more in parallel instead of sequential): During the phase of active automation the drivers’ state and their level of being out-of-the loop influences the readiness for takeover. In the moment of the takeover request the driver starts taking up information and processing it, which includes to firstly perceive the takeover request itself and to subsequently build up an adequate situation awareness. These processes of action preparation serve as a basis for the decision on the adequate reaction in the specific situation. The last step is the initiation of an observable behavior for taking over the driving task from the system which results in a measurable performance outcome. As the method was explicitly designed to map the four steps in this takeover process Perception - Awareness – Decision – Action it is called PADA-AD Test for automated driving (AD=Automated Driving).

## Method details

In the following chapter, detailed descriptions are given how the method should be applied. These include specifications of

• The test course (including the generic layout of a test section, variations of the takeover scenarios, the arrangement of the tests sections towards a complete test course and possible adjustments).
• The implementation of the investigated automated system and HMI.
• The non-driving-related task (NDRT) to be performed during the automated drive.
• The dependent measures to be assessed.
• The instructions given before the test.
• The characteristics of the test sample.
• The equipment needed to conduct the test and the measurements.

### Test course and test scenarios

The test should be conducted in a driving simulator. The test course consists of a three-lane highway with 12 test sections where the driver repeatedly has to take over control from the automated system. Lane width is defined with 3.75 m for all three lanes (plus a hard shoulder with 2.5 m width at road boundaries). The surrounding consists of reduced planting with few trees or bushes in the periphery.

#### Generic layout of a test section

All test sections are similar regarding the layout of the road and the environment but differ in the included takeover scenario. Each section of 2 km length first contains a phase where the driver drives with the automated system and is then faced with an obstacle on the test vehicle's lane. The obstacle serves as a system limit and as a reason for taking over the driving task from the automated system. In case of a Level 3 system, a TOR is issued at a certain time before the obstacle on which the driver has to react. After passing the obstacle, the driver is requested to drive manually until the end of the section. The first part of the road can be slightly curvy, however in the moment of the TOR as well as before and after the obstacle the road layout should be straight.

At the start of each section (at route meter 10), the system has to be activated. This can be either performed by the drivers themselves or can be triggered automatically. The automated activation prevents from potential usability problems with system activation and ensures that each driver drives for a constant amount of time with the active system. If this implementation is not possible for technical reasons, or if the usability of system activation is of interest, the drivers can also be instructed to activate the system themselves.

After activation, the system automatically adapts the speed to 100 km/h and drives on for a duration of approximately 45 s. During automated driving, the driver should continuously engage in a specified non-driving related task (NDRT). This is to ensure that the driver is both visually and cognitively out of the loop before a takeover situation.

At route meter 1300 within the test section, the TOR is issued. At this time, the obstacle suddenly appears on the test vehicle's lane in a distance of 221 m ahead (i.e. at route meter 1521). The obstacle consists of four red-white striped safety beacons positioned across the complete width of the lane, so that it is necessary to execute a complete lane change in order to avoid a collision with the obstacle. The distance of 221 m between the TOR and the obstacle results from the aimed time budget of 8 s until the vehicle will collide with the obstacle if the driver would not intervene. This time window was defined as suitable in order to give the driver enough time to take situation-aware decisions on the adequate behavior in the takeover situation. When investigating automated systems with other defined set speeds the distances between the TOR and the obstacle have to be adapted in order to achieve the 8 s time budget accordingly. The drivers have been instructed in advance to solve the situation in such a way that no one is endangered.

The test sections differ in various factors which define the adequate takeover reaction in order to resolve the given situation without an accident (see next chapter for further details on the scenario variants). One of these factors is whether a vehicle on the target lane approaches from behind at a higher speed in the takeover situation or not. If this is the case, the drivers must reduce their speed by braking before executing the lane change in order to avoid colliding with the obstacle and to allow the rear vehicle passing before they can safely change lanes.

To ensure that all drivers have a comparable initial level of situational awareness at the moment of the TOR, drivers should be prevented from seeing the scenario prematurely by control glances during automated driving, even if they are distracted by an NDRT. For this reason, both the obstacle and the traffic behind - if present - only pop up in the driving scene or, in the case of traffic, in the rearview mirrors at the moment of TOR.

**Fig. 2:**
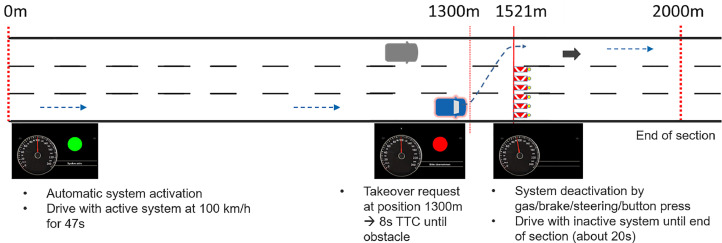
Schematic sequence of one test section with a length of 2000 m. Blue vehicle: subject vehicle, gray vehicle: rear vehicle, blue dotted line: intended driving trajectory of the subject vehicle.

After passing the obstacle, the driver is instructed to stay on the current lane in case of a lane change and drive manually until the end of the test section at route meter 2000. To help the drivers determine whether they are in the correct lane, an arrow marking is placed on the destination lane. Fig. 2 shows the sequence of a test section with an exemplary HMI as used in the validation studies.

#### Variants of the takeover scenarios

The entire test course is made up of 12 sections containing 12 variants of the takeover scenario. The variants differ in the following factors, each of which is intended to represent different demands on the driver:

• Presence of an obstacle: yes vs. no (8 vs. 4 situations): Due to the presence or absence of an obstacle in the subject vehicle's lane the driver has to decide whether a lane change is necessary or not.
• Direction of the lane change: left vs. right (4 vs. 4 situations with obstacle): By varying whether the subject vehicle starts the test section driving on the left, middle or right lane, the driver has to decide for the correct direction of the lane change.
• Number of necessary lane changes: one vs. two (4 vs. 4 situations with obstacle): By blocking either only the subject vehicle's lane or additionally the neighboring lane, the driver has to decide whether a single lane change or a dual lane change is necessary and has to execute that maneuver.
• Need for an additional braking maneuver: yes vs. no (4 vs. 4 situations with obstacle): Due to the presence of additional rear traffic the driver has to decide whether a braking maneuver is necessary or not, and how strong it has to be.

The factor of whether a lane change has to be carried out at all is intended to prevent drivers from learning that they always encounter an obstacle as a takeover reason and thus automate their behavior. Therefore, four so-called distractor scenarios were introduced in which the drivers receive a takeover request but do not have to change lanes. These distractor scenarios can be filtered out subsequently from most of the conducted data analyses. If there is a special interest in examining TORs caused by a malfunction compared to TORs due to road-based system limit (together with specific HMI outputs for those scenarios), these scenario type can be analyzed separately from the other scenario types.

To create even less predictability, there are four variants of these distractor scenarios:

• Variant 1, in which there is neither an obstacle, nor a vehicle in the rear.
• Variant 2, in which there is an obstacle but in a different lane and no vehicle in the rear.
• Variant 3, in which there is a vehicle in the rear but in a different lane and no obstacle.
• Variant 4, in which there are both an obstacle and a vehicle in the rear but both in different lanes.

In the scenarios with additional rear traffic, the approaching vehicle is 20 m (in the case of a single lane change) or 50 m (in the case of a dual lane change) behind the test subject vehicle on the target lane at the time of the TOR. The approaching speed of the vehicle is 113 km/h or 130 km/h, respectively. The choice of the appropriate distance and relative speed in both cases was determined in preliminary studies and aimed at creating a situation in which the driver has to brake compulsorily in order to avoid a collision with the obstacle and the rear traffic. Solving the situation by merely swerving should not be possible here, in contrast to the situations without rear traffic. It should also not be possible to increase the speed in order to pass the obstacle before the approaching vehicle.

In detail, the following scenario variants were selected for the test setup (see Table 1). The selection of the variants was determined in pre-tests according to a comparably moderate level of assessed situation complexity (see results from study P1 in the *method validation* chapter).

**Table 1:** The 12 variants of the takeover scenarios used in the test setup with a schematic illustration of the situation and names used for the variants. Abbreviations: “M “= middle lane,” L” = left lane; “R” = right lane; “→” = direction of the lane change;” _0″ = without rear vehicle,” _1″ = with rear vehicle, “LC” = lane change.

Type of scenario	Takeover scenario variant	
Distraction scenarios	No obstacle/no rear vehicle: L_0	Obstacle/ no rear vehicle: M_0_H
	No obstacle/rear vehicle on irrel. lane: L_1	Obstacle + vehicle on irrel. lanes: M_1_H
Single LC without rear vehicle	Middle lane to right lane: M→R_0	Middle lane to left lane: M→L_0
Single LC with rear vehicle	Left lane to middle lane: L→M_1	Right lane to middle lane: R→M_1
Dual LC without rear vehicle	Left lane to right lane: L→M→R_0	Right lane to left lane: R→M→L_0
Dual LC with rear vehicle	Left lane to right lane: L→M→R_1	Right lane to left lane: R→M→L_1

#### Complete driving course

**Fig. 3:**
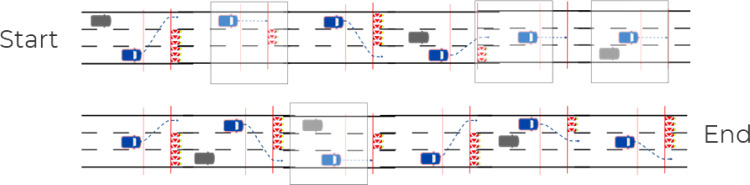
Test course with the sequence of the 12 scenario variants. Blue vehicle: subject vehicle, gray vehicle: rear vehicle, marked scenarios: distractor scenarios without the need to change lanes.

By combing the 12 test sections into a meaningful sequence, a driving course of about 15 min duration results, which can be driven through continuously, and in which the driver must repeatedly decide on the correct action due to the different combinations of the various situational factors in each scenario. Fig. 3 shows the recommended sequence of scenario variants as result from the validation studies. In case the test course should be repeated, e.g. while driving with a second HMI variant, another sequence can be generated out of the 12 scenarios (e.g. by simply mirroring the initial lanes of the scenarios). If one wants to have the worst case as the first contact, it is recommended to choose a scenario with rear traffic as first scenario.

#### Possible adjustments on the test course

Adjustments on the number and frequency of the included scenarios can be made dependent on the specific research question. In case the initial contact with the system's HMI is of particular importance, the number of scenarios can be reduced. Additionally, it is possible to increase the length of the automated drives in between the takeover scenarios from currently one minute to longer time intervals. This might be suitable for driver state related research questions (e.g. whether a driver who has been driving in automated mode for a longer period of time reacts differently to a takeover request than someone who has only been shortly driving in automated mode). In order to generally reduce the predictability of the takeover request, the time intervals between takeover situations in which automated driving takes place can also be deliberately varied in length.

### Implementation of the investigated automated system and HMI

The automated system which is subject of the investigation should be implemented in the driving simulation. Optimally, all relevant states of the system to be tested, including the transitions between these states, their associated functionality and all relevant HMI displays, should be mapped as best as best as possible. This must include at least an automated longitudinal control function that maintains both a defined distance to the lead vehicle and a specific set speed as well as an automated lateral control function that additionally provides the driver with support in lateral guidance. In addition, the system must be able to respond to driver input. This includes at least the driver-initiated activation of the system via a corresponding control element as well as the possibility to take over vehicle control by the driver either via the control element, the steering wheel or by actuating the brake.

In addition, the implemented HMI should give feedback on the current system state as well as on driver- or system-initiated changes between system states (e.g., the takeover request at L3 system limits). The HMI should include the system's visual display concept, i.e., a visual display in the driver's field of view (usually the instrument cluster or a head-up display) and, if necessary, displays at other locations, such as displays in the center console or in the steering wheel. Ambient lighting, which can provide feedback on the system state, might be also part of the HMI concept. In addition to the visual modality, acoustic feedback is also common, such as tones or voice output in case of system warnings or notifications, as well as haptic signaling devices such as seat vibration. System/HMI-specific aspects that are assumed to be less important for the questions to be investigated, do not necessarily have to be implemented.

### Non-driving related task (NDRT)

In order to determine the influence of the out-of-the-loop condition on the driver's state and thus the ability to takeover, a non-driving related task (NDRT) should be implemented. This should ensure that all drivers experience the takeover situation under comparable conditions (e.g. with regard to the degree of out-of-the-loop).

**Fig. 4:**
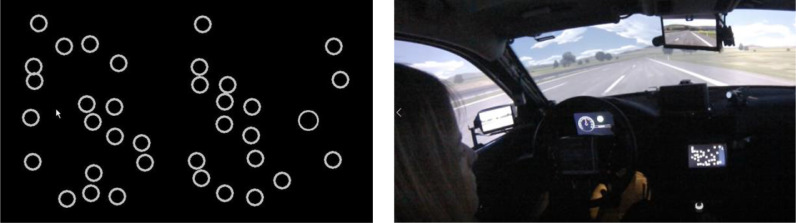
The SURT and its exemplary implementation in the WIVW driving simulator.

The Surrogate Reference Task (SuRT; [8]) is suggested as a suitable secondary task. In this task, subjects see a series of circles on a display (see Fig. 4). One of them is slightly larger than the other circles. This circle must be selected. The task should be presented at a location that forces the drivers to avert their gaze completely from the driving task so that they are no longer able to perceive the road peripherally, i.e., optimally at a position in the lower area of the center console. The input medium can be a rotary pushbutton which can be used to move a selection area to the left and right and to select the area by pressing it. If an input is confirmed, the next task appears. The SuRT is available in different difficulty levels (regarding the size of the distractors and the number of selectable areas). A detailed description of the task can be found in the ISO Norm [9]. In the studies presented here, the SuRT was used on the medium difficulty level.

The advantage of this artificial task is that it can be standardized, controlled, and varied in its demands on the driver. The resources claimed are visual-motoric in nature to produce the greatest possible interference with the takeover of vehicle control. The preliminary studies and validation studies for the development of the method showed that the drivers’ task involvement in this task was very high.

To ensure that the desired level of distraction is reliably generated at the moment of the takeover request, the subject is instructed to perform the task continuously during the automated drive. If the duration of the automated drive is extended from the current approx. 45 s to a longer period, it may be useful to provide the NDRT only in certain phases in order to avoid fatigue effects. It should then be mandatory to start such a phase before the takeover request to ensure that the driver is engaged with the NDRT at that time. In order to additionally increase the motivation to engage in the task, the number of correctly completed trials can be reported back to the subjects. The drivers’ performance in the NDRT is not further analyzed.

### Dependent measures

The parameters listed in Table 2 should be recorded in order to analyze the takeover performance in the test scenarios. The table lists all measures together with a definition and the recommended analyzed parameters. In addition, an attempt is made to categorize on which level of information processing within the cognitive takeover model (see Fig. 1) the analysis is focused on (however, these categories might not be fully independent and sometimes mixed up).

• Focus on perception-related aspects: How fast is the TOR perceived? To what extent does the HMI direct driver's attention to the takeover scenario?
• Focus on situation awareness and decision-related aspects: To what extent does the TOR/HMI support the driver in the assessment of the situation and in the decision for an adequate reaction?
• Focus on action-related aspects: How well is the action performed by the driver? To what extent is the HMI able to avoid safety-critical-performance outcomes?

**Table 2:** List of dependent measures to be assessed in the test procedure categorized in types of variables with definition, recommendation for analyzed parameters and focus of analysis.

*Measure*	*Definition*	*Analyzed parameter*	*Focus of analysis*
*Takeover performance measure with the focus on reaction speed*			
Takeover Reaction Time [s]	Mean value across test course, excluding distractor scenarios	Time from TOR until the first reaction that deactivates the system	Compare HMI variants Perception
Brake Reaction Time [s]	Time from TOR until brake pedal position > 10% of max. is reached		Compare HMI variants Awareness and Decision
Steering Reaction Time [s]	Time from TOR until steering angle > 2° is reached		
Hands-on Reaction time [s]	Time from TOR until hands are touching the steering wheel		Compare NDRT (e.g. handheld devices); hands-on vs. hands-off driving Motor-Readiness and Perception
*Takeover performance measure with the focus on reaction quality*			
Type of takeover	Type of first reaction leading to deactivation of the system: braking, steering, accelerating or pressing the button, others	Frequency of cases across test course (or across scenario types); including distractor scenarios	Compare HMI variants Awareness and Decision
Max. long. deceleration [m/s²]	Maximum value in the time interval from TOR until reaching the obstacle	Mean value across test course; excluding distractor scenarios	Compare HMI variants Action-Execution
Max. lateral acceleration [m/s²]			
Minimum TTC or Time Headway to the obstacle [s]	Value at the moment when the subject's vehicle leaves own lane with center of gravity in relation to obstacle		
Standard deviation of lateral position [m]	Value in the time interval from the moment passing the obstacle until 10 s afterwards		
Number of (near) collisions with obstacle	Complete crash with the obstacle or near-crash (defined if TTC < 1 s)	Frequency of cases across test course (or across scenario types); excluding distractor scenarios	Compare HMI variants Action Success Absolute safety-relevant evaluation of HMI
Number of (near) collisions with rear-end traffic	Complete crash with the rear vehicle or near-crash (defined if TTC < 1 s)		
*Eye Tracking measures*			
Location of First Glance away from NDRT	Area of interest of the first glance away from NDRT: HMI vs. road	Frequency of cases across test course (or across scenario types); including distractor scenarios	Compare HMI variants Perception
Location of First Glance in Mirror	Type of mirror where the first mirror glance is directed to: left, rear, right)		Compare HMI variants Situation Awareness
Road Glance Reaction Time [s]	Time from TOR until first glance is directed away from NDRT to road	Mean value across test course; including distractor scenarios	Compare HMI variants Perception
Mirror Glance Reaction Time [s]	Time from TOR until first glance is directed in a mirror		Compare HMI variants Situation Awareness
*Subjective measures*			
Subjectively perceived situation criticality by using SCA [9]; [0…10] rating	Online directly after each takeover scenario; during the phase of manual driving	Mean value across test course (or across scenario types); including distractor scenarios	Compare HMI variants (global rating)
Subjective evaluation of the HMI/system	Various measures of user experience, system usability, etc. after test drive	Single rating for each HMI variant	
Evaluation of specific design aspects	Various measures of specific HMI aspects, after test drive		Compare HMI variants
Subjective evaluation of driver-state related aspects	Various measures of workload, attention, drowsiness etc. after test drive	Single rating across the complete drive	Compare HMI variants, durations of automated driving, countermeasures on distraction/ drowsiness

The focus is mainly on a relative comparison of HMI variants based on mean values across the complete test course. In most cases, distractor scenarios should be excluded from the analyses. The number of collisions can also be used as absolute safety-relevant evaluation of a system or HMI.

For assessing the subjectively perceived criticality of the takeover situation, the Scale of Criticality Assessment SCA is recommended [10]. This is an eleven-point single item scale. The rating procedure has two stages: The drivers are first to decide on one of the five verbal categories: ‘imperceptible’ (0 pt.), ‘harmless’ (1–3 pts.), ‘unpleasant’ (4–6 pts.), ‘dangerous’ (7–9 pts.) and ‘uncontrollable’ (10 p.). They can then fine-tune their rating by making a numerical judgment. The drivers are supposed to assess the situations concerning the necessary compensatory effort. For precise instructions on the usage of the scale see [9].

Aspects of user experience, acceptance or system usability can be queried by means of standardized questionnaires, such as the UEQ (User Experience Questionnaire [11]), the Van der Laan Scale ([12]) or the System Usability Scale (SUS [13]). Questions about specific design aspects of a system or HMI should be self-designed. To control for methodological aspects, questions can be asked about the developed fatigue (using the Karolinska Sleepiness Scale KSS [14]) before and after the test drive, the perceived workload during the drive, and the involvement in the NDRT.

As the NDRT is merely used as a methodological mean to create the out-of-the-loop phenomenon (together with the popping up of the situation the driver has no chance to anticipate the system limit) an analysis of the NDRT performance is not part of the analysis of the takeover performance. The NDRT performance could be used to check whether the method to control the driver being-out-of-the-loop worked in the intended way.

### Instructions

Before starting the test drive, the driver should be informed about the aim and the procedure of the study in a generic way without any detailed information about the design of the HMI. If subjects are not yet familiar with the driving simulator, a manual familiarization drive of 5 to 10 min should take place, where drivers can experience the visual impression in the simulator and the motion feedback in the case of steering and braking maneuvers. This drive can also be used to determine susceptibility to simulation sickness. If drivers are already familiar with the driving simulator, the familiarization drive can be skipped.

This is followed by instructions on the basic functionality of the system. In the case of an L3 system, this contains the information that the system takes over lateral and longitudinal control of the vehicle, so that the drivers do not have to accelerate, brake or steer anymore while the system is active. The system will keep them in the lane and maintains a constant speed. In addition, the drivers are told that the system will automatically activate at a certain point at the route (if automatic activation is used). If the drivers have to carry out the activation for themselves, the necessary control elements and operating steps are explained. Furthermore, they are instructed to take their feet off the pedals and their hands off the steering wheel while driving with the active system. The drivers are also informed that this level of automation does not longer require to permanently monitor the driving task, but allows to engage in other activities. After that, instructions on how to perform the SuRT follow. The drivers should perform the task permanently during automated driving at their own pace.

Furthermore, they are told that it may happen that the system cannot handle certain situations and therefore prompts them to take over the driving task. In such a case they will receive a takeover request consisting of a visual notice in combination with another modality. Information on how the driver can take over the driving task in the event of a takeover situation, e.g., by braking, accelerating, steering, or pressing a button, should be provided. Information about the nature of the takeover situation should include that it will be necessary to change lanes in some cases, but not always.

The question whether a practice drive with the system should be included before the actual measurement drive, and what its content should be, is dependent on the research question. In the studies conducted during method development, different approaches were taken. If the focus is on intuitive understanding of the takeover request, there should be no practice drive. Otherwise the practice drive could be used to let the drivers experience the takeover situation and the associated takeover request.

If the method is applied to an L2 system, the drivers must be informed that such a system only assists with lateral and longitudinal control and that they therefore remain responsible for driving and monitoring the traffic environment. Frequently, the driver must also keep their hands on the steering wheel.

### Test sample

The correct way to define the sample size required for HMI questions is to calculate it in advance according to the aimed effect size (for an overview of sample size calculation see [15]). However, this approach leads to a rather high number of required subjects. In practice, for economic reasons, a number of at least ten subjects per experimental condition has proven to be a reasonable and efficient solution. For example, if the study aims to compare two HMI variants, at least 20 subjects should be tested.

Gender of the sample should be equally distributed. An equal distribution of age among four age groups (group 1: 18–24 years, group 2: 25–39 years, group 3: 40–54 years, and group 4: >55 years) should be aimed for, as recommended by the National Highway Traffic Safety Administration; NHTSA [16]). It should be taken care that all subjects have a comparable level of prior experience with assistance and automated systems. Since it cannot be assumed that at the current stage of development of automated systems all drivers have prior experience with L2 or L3 functions, at least all drivers should have experience with L1 systems, i.e. Adaptive Cruise Control. This ensures a certain level of prior knowledge about the general functional capabilities and operation of such systems.

### Test equipment

The test should be conducted in a driving simulator. If possible, a simulator with a motion system should be used as receiving motion feedback is particularly important for a realistic evaluation of the situation criticality of takeover situations in automated driving. [17] recommend that all studies with takeover situations should be conducted with a motion system, since motion feedback influences numerous subjective and objective takeover criteria. In terms of HMI content, motion feedback appears to be particularly important when haptic or kinesthetic feedback, e.g., a brake jerk, is to be investigated in conjunction with TOR. In addition, motion feedback enhances the degree of immersion, especially when the driver is engaged in visually distracting NDRTs, which reduce visual feedback from the driving environment.

**Fig. 5:**
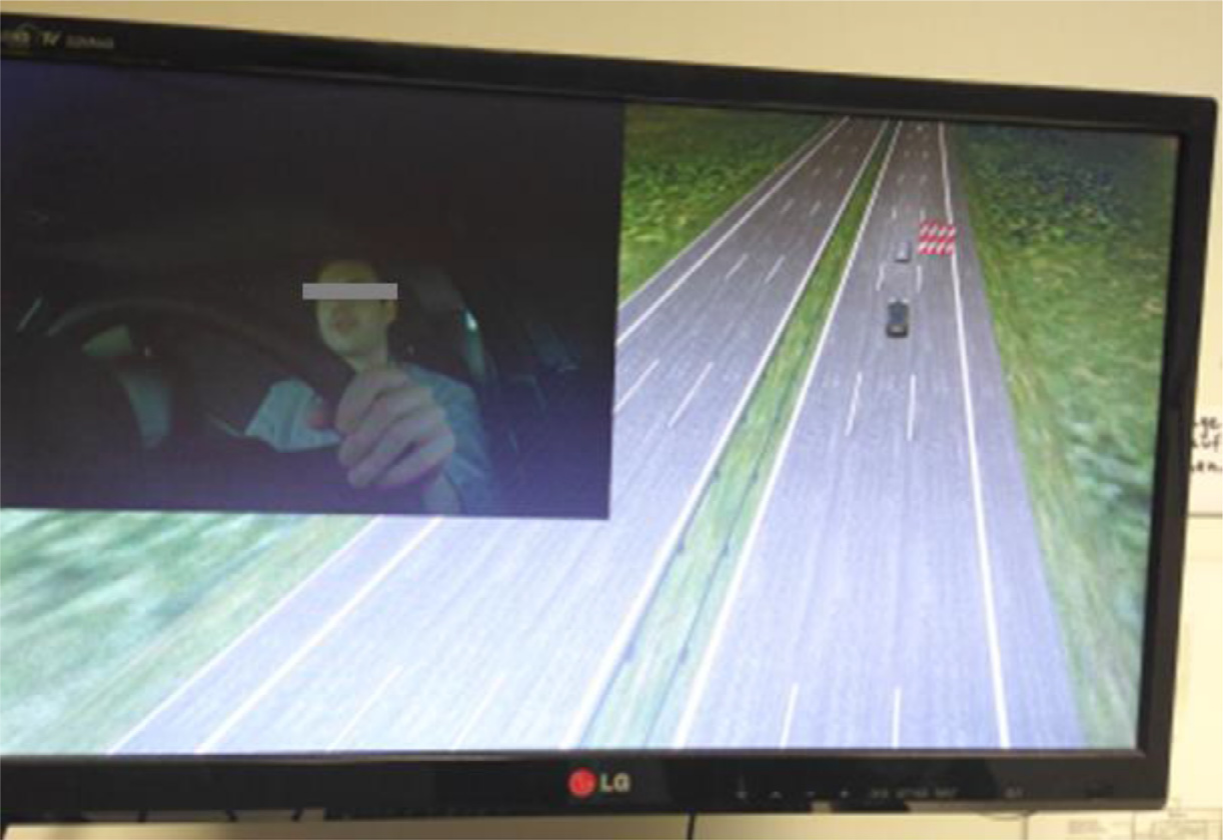
Online observation of the driver and the driving scene via monitor.

Since hands-on reaction time might be an interesting parameter for the evaluation of the takeover performance at L3, a hands-on detection should be integrated in the steering wheel. If not available, this data can also be inferred from a subsequent video analysis. For assessing driver's glance behavior (e.g. in order to explore driver's acquisition of situation awareness in reaction to the TOR), it is necessary to implement an eye tracking system. This should be able to assess reaction times of glances to certain glance areas (e.g. road ahead, cluster display, NDRT) as well as distribution of glances to these areas. In order to observe drivers’ interaction with the system or the NDRT, the experimenter should be able to watch the driver as well as the driving scene online during the conduction of the test (see Fig. 5).

In addition to the collected objective driving data, a protocol sheet is recommended in which the investigator can enter special observations during the test drive for each scenario. On the one hand, this can be used to explain conspicuous cases in the data analysis. On the other hand, the data can describe certain aspects of the driver's behavior that cannot be explained by the analysis of driving parameters, but can provide possible additional value in the interpretation of the results: For example, it should be protocolled when the driver changes lanes without necessity in the distractor scenarios. Special remarks made by the driver can also be entered in the protocol sheet.

In addition, it can be used to record the subjective criticality judgments made after each takeover situation. In order to facilitate the usage of the SCA rating scale [10] for the driver during the manual phase of the test sections, the rating scale can be mounted on the steering wheel. Other test materials, e.g., a follow-up survey after the single test drives or after the entire study, must be prepared according to the respective study question.

### Method validation

In order to validate the developed test method a number of preliminary tests and validation studies had been performed in the WIVW driving simulator. The basic layout of these studies is described in the following chapter. Afterwards, the main results of the studies are summarized.

### Basic layout of the validation studies

**Fig. 6:**
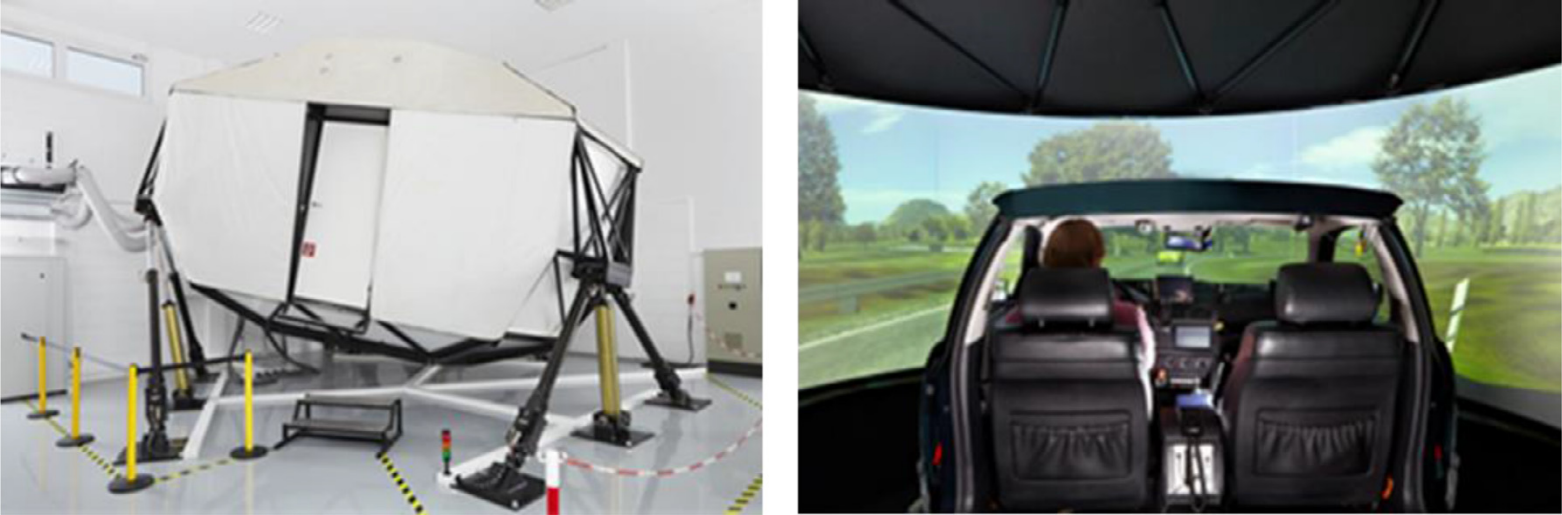
The motion-base driving simulator of WIVW GmbH.

All validation studies and pre-tests took place in the dynamic driving simulator of WIVW GmbH (see Fig. 6).

A prototypic automated system combining automatic distance and speed control with automatic lateral guidance was used for the studies. The system's availability depends on certain conditions, such as certain road sections and a centered position in the lane. In the described tests, the system activated automatically when the preconditions were met. If, at the moment of automatic activation, the driver was not driving centrally enough in their lane and automatic activation therefore failed, the system could also be activated manually by the driver. A button on the left side of the steering wheel was used for this purpose in the studies. In the studies described, the set speed was automatically set to 100 km/h. While the system was active, the driver could override the system via the accelerator pedal without deactivating it. Even if the driver put their hands on the steering wheel, the system remained active. The takeover request was issued depending on the position of the route (here at route meter 1300 in each test section). The system remained active until the defined takeover time had elapsed and continued to drive at the constant speed or until the moment when the driver took over control of the vehicle. Takeover could be performed either by actuating the brake or the accelerator pedal, by entering a steering input above 2°, and by pressing a button. Bringing the hands to the steering wheel was not sufficient. If the driver did not take over within the specified takeover time, the system switched itself off.

**Fig. 7:**
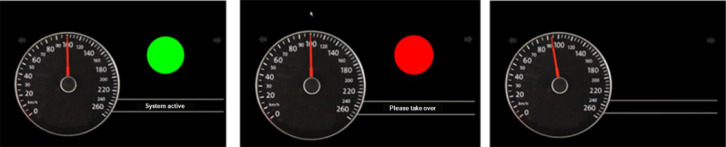
Prototypical status display of the automated system in the instrument cluster display used in the validation studies.

A simple prototypical display was used to indicate the system status (see Fig. 7). Right beside the speedometer in the instrument cluster display, a colored circle was shown in combination with a text display. In the active state, a green circle with the text “system active” was displayed, the takeover request was signaled via a red circle with the text “please take over” together with an acoustic signal. The status display was empty when the system was inactive.

**Fig. 8:**
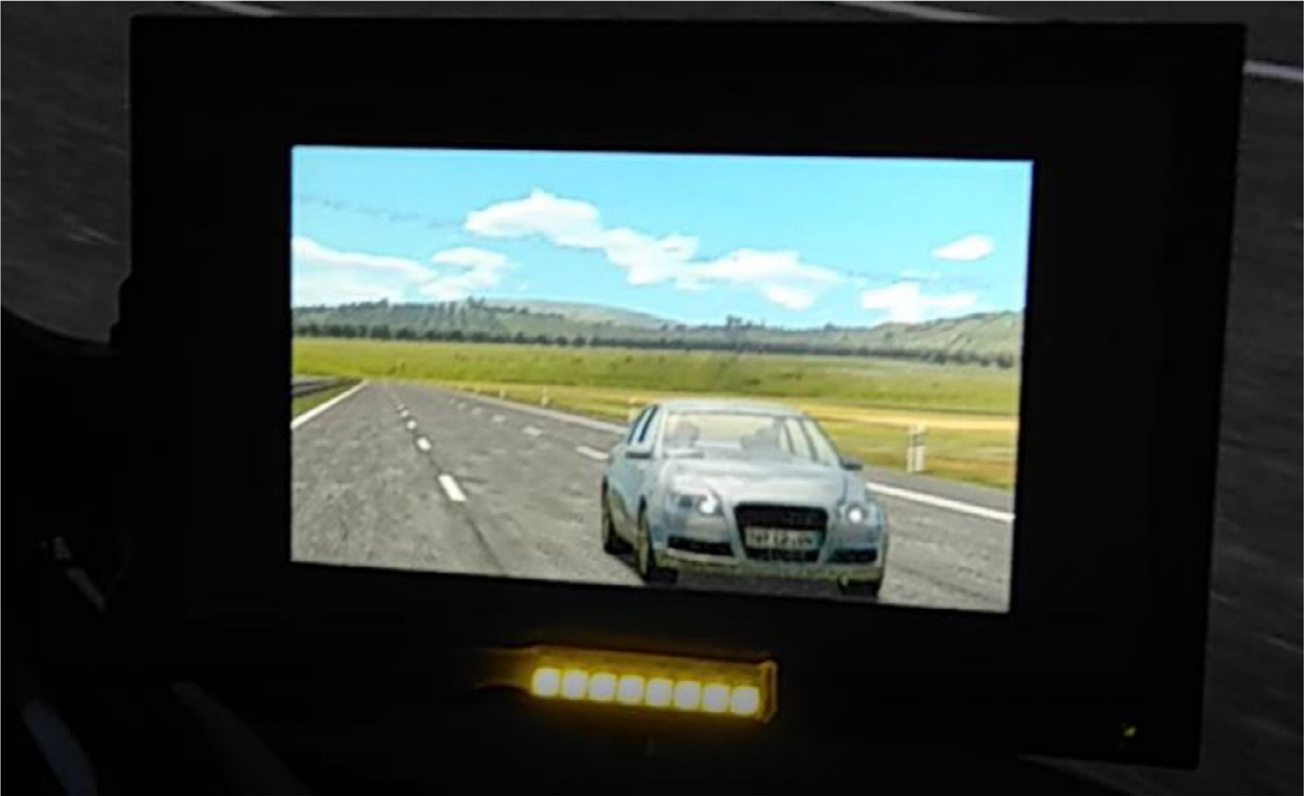
LED warning in the exterior mirror for rear traffic as exemplary HMI variation used in validation study 1 and 2.

With the aim of evaluating the extent to which the method is sensitive for differing system or HMI variants with regard to their effects on the driver's ability to takeover, two different display concepts for TORs were prototypically implemented and examined in the validation studies. In the two pre-tests, the acoustic feedback at the moment of the TOR was varied: In one condition, an urgent warning tone was issued, in the other condition a softer repeated chime. In validation studies 1 and 2, in addition to the urgent warning tone, an LED in the respective exterior mirror warned the driver of relevant rear traffic in the corresponding neighboring lane in the manner of a blind spot warning in one condition, but not in the other (see Fig. 8).

### Main results of the studies

The following chapter describes the main results of five simulator studies that were conducted with the goal of method development and validation: Two preliminary studies and three validation studies. Table 3 gives an overview of the number of subjects, the central questions and the main results or decisions of the studies. Due to the large number of investigated studies and the analyzed data it is not possible to show all of them here in detail. For a more detailed overview of all results from the performed validation studies see [1].

**Table 3:** Overview of conducted preliminary and validation studies in the driving simulator.

Study	*N subjects*	*Central question and study design*	*Main results or decisions*
P1	*N* = 6	Is the test course able to detect differences between HMI variations? Which test scenarios are sensitive? Driving with L3 system; Comparison of two distinct takeover sounds (urgent vs. soft) in a within-subject design	HMI differences had effects on takeover performance (esp. reaction times); Methodological decisions: reduce number of scenarios, increase number and variation of distractor scenarios, remove sound of surrounding traffic, adjust setup position of rear traffic
P2	*N* = 10	What were the effects of methodological adjustments compared to P1? Driving with L3 system; Comparison of two distinct takeover sounds (urgent vs. soft) in a within-subject design	Methodological adjustments led to comparable HMI effects on takeover performance in comparison with P1; fewer number of braking events in scenarios without traffic; increased braking response times as a result of later perception of rear traffic
V1	*N* = 20	Is the test procedure capable of mapping HMI differences related to situational awareness? Driving with L3 system; Comparison of two distinct HMI variations: with vs. without additional LED lights in the mirrors warning from rear traffic from behind in a within-subject design	Test is suitable for revealing specific HMI differences that aim to increase the driver's situation awareness: Faster takeover times were shown only in situations with traffic when an additional warning was given by means of an LED in the exterior mirror; no HMI effects on takeover quality and subjective situation criticality
V2	*N* = 20	What influence does the time budget of the takeover request have on the results of test procedure? Driving with L3 system; Comparison of results between V1 (8 s time budget for takeover) and V2 (5.5 s time budget for takeover)- using the same HMI variation compared to V1	Reducing the time budget from 8 s to 5.5 s makes the takeover scenarios more critical overall, forcing drivers to react faster and more strongly. However, drivers have to react so quickly that they may not have time enough to build up situation awareness; decision to choose 8 s time budget for the final test setup
V3	*N* = 30	Is the test procedure suitable for the evaluation of L2 systems (for example investigating DMS strategies)? Driving with L2 system; Comparison of a state-dependent strategy to keep driver in the loop (Driver Monitoring System DMS observes whether driver looks away from road > 4 s and issues warnings) with a situation-dependent strategy to bring the driver back in the loop (Monitoring Request to increase monitoring behavior due to an unclear traffic situation 10 s in front of the obstacle) with a condition without any interventions by the system- using a between-subjects design	Descriptively, shorter gaze reaction times until perception of the situation and shorter intervention times depending on the Driver-in-the-loop strategy (with vs. without intervention). However, effects were overlaid by strong individual differences in monitoring behavior during automated driving, so that none of these differences reached statistical significance. In the condition without intervention, critical situations with the obstacle occurred more frequently or, conversely, such situations could apparently be successfully avoided by the interventions.

#### Preliminary study 1 (P1)

The goal of P1 was to evaluate whether the test course was able to detect differences between HMI variations and which test scenarios were especially sensitive for HMI differences. In contrast to the final test course the test course in this preliminary study still consisted of 15 scenarios:

• Three distractor scenarios without obstacle, in which no lane change was necessary (test vehicle is either on the left, center or right lane).
• Eight single lane changes (from left to center with vs. without traffic, from right to center with vs. without traffic, from center to left with vs. without traffic, from center to right with vs. without traffic).
• Four dual lane changes (from left all the way to right with vs. without traffic, from right all the way to left with vs. without traffic).

The HMI used was the one described in the chapter above for the two pre-studies: the type of acoustic feedback during the TOR was varied as within factor (urgent vs. soft takeover sound in two subsequent drives). SuRT was used as the non-driving related activity. *N* = 6 subjects participated in the study, four of them female (employees of the WIVW; due to the pandemic situation at that time in spring 2020, no external subjects were allowed to participate in the study).

The analysis showed that the differences in the acoustic takeover sound led to a different perceptibility of the TOR and thus to different reaction times. This in turn influenced other parameters: In the condition with the soft chime, longer braking reaction times together with stronger decelerations were measured, which led to comparable minimum TTCs to the obstacle compared to the condition with the more urgent warning chime. The longer brake reaction times also required stronger steering maneuvers, which were accompanied by stronger lateral accelerations and thus stronger problems in lane keeping after passing the obstacle.

The test setup was able to generate different behavioral responses depending on the situation. However, braking was also observed in situations where it was not actually necessary. It was probably used as the simplest and most efficient method of system deactivation. The cognitive difficulty of the scenarios, which was queried online after each scenario, distinguished four categories of scenarios: very easy scenarios (the distractor scenarios), easy scenarios (the scenarios with single lane change from left/right to center without traffic), moderately difficult scenarios (single lane change from left/right to center with traffic, single lane change from center to left/right without traffic, dual lane change with and without traffic), and difficult scenarios (single lane change from center to left/right with traffic). The scenarios in the moderate category (eight scenarios) were found to be the most appropriate for showing differences between the HMI variants.

For this reason, and due to the fact that high learning and expectancy effects occurred, it was decided to reduce the set of scenarios from 15 to 12, focusing on the scenarios of moderate cognitive difficulty with high discriminatory power. To further reduce predictability, the number of distractor scenarios was increased and made more variable. In addition, it had turned out that the sudden appearance of the surrounding traffic had acted as an additional acoustic cue to recognize that braking was necessary. Furthermore, the vehicle approaching from behind in the neighboring lane was already visible in the side window at the moment of the TOR, so that the subjects did not necessarily have to look in the mirrors. Both aspects resulted in relatively frequent and quick braking maneuvers. To counteract these effects, both the setup position of the traffic was adjusted (the rear vehicle was placed further back) and the acoustic cue of the surrounding traffic was removed.

#### Preliminary study 2 (P2)

This preliminary study was conducted with the aim of testing the effectiveness of the optimizations defined in the previous study and to make the findings statistically verifiable on the basis of a larger sample. The test set-up was therefore identical to that in P1, apart from a few adjustments: Instead of the 15 scenarios, the test course now consisted of twelve scenarios (the ones defined in the final test protocol). The HMI variants investigated were identical to P1. SuRT was again used as NDRT. This study involved *N* = 10 external subjects (5 female) recruited from the WIVW test driver panel. The mean age was 36 years (min. 24, max. 46; SD = 8.1 years).

In summary, the following conclusions can be drawn from the study: The predictability of the scenarios was somewhat reduced by the methodological adjustments. The HMI differences found in P1 were also evident in P2 in that the lower urgency of the HMI tone caused later hands-on times, later glances back to the road, later takeover times, and a tendency for later steering responses compared to the HMI variant with urgent warning tone. As expected, the frequency of braking was now significantly reduced in scenarios without traffic. The initial reaction here was now predominantly steering. In particular, braking response times were now significantly longer compared to P1. This indicates that the perception of rear traffic was now more difficult, which is why drivers took longer to start the necessary braking (a necessary mirror glance now occurred significantly more often before braking was initiated). Overall, it can be noted that the test set-up is able to reveal differences between HMI variants that are characterized by different perceptibility of the takeover request (e.g., different acoustic takeover signals). In addition, the situations generated different types of behavioral responses, speed of responses, and quality of responses. It was decided that the basic principle of the test setup can remain in its present form.

#### Validation study 1 (V1)

Validation study 1 (V1) investigated whether an HMI providing the driver with additional information about the takeover situation offers advantages over an HMI only conveying the need to take over. Several studies showed that adding information to the TOR can increase the drivers’ situation awareness and assist them in the takeover response. Knowledge about the presence or position of an obstacle or additional surrounding traffic, the requested action, etc., might enable a faster, more effective, higher-quality takeover (e.g. [18], [19], [20], [21]). So-called "ambient lighting" concepts proved to be useful display concepts in order to quickly and efficiently direct driver attention and support situation interpretation (e.g.: [18,[22], [23], [24], [25]]). To address this issue, the following HMI concept was investigated in this study: An additional LED in the exterior mirror warned the driver from approaching rear traffic when changing lanes. The drivers still had to pay attention to the scenery in front, e.g. to observe which lane is blocked. The hypothesis was that drivers with LEDs would brake faster in scenarios with relevant traffic and would be less likely to brake unnecessarily in scenarios without traffic.

For this study, the same test setup as in P2 was used with a total of 12 scenarios (four distractor scenarios and eight lane change scenarios). The basic HMI of the pre-studies was now enriched with the additional LED warning in one of two HMI conditions (with vs. without LED). For this purpose, a yellow flashing LED bar was stuck to the lower edge of the two outside mirrors (see Fig. 8). The LED bar flashed at the moment of the TOR for a duration of 3.2 s (as long as the vehicle was visible in the mirror) with an on-time of 150 ms and an off-time of 200 ms, 9 times in succession. The LED warning was only issued when traffic was in a lane relevant to the drivers, i.e. when they had to change to this lane. For the scenarios used in V1, this means that the warning was issued in a total of four of the 12 scenarios. In the distractor scenarios, no warning was issued because the traffic presented there was irrelevant for the driver, since they did not have to change lanes.

SuRT was again used as NDRT. The sample consisted of *N* = 20 subjects (*n* = 10 female) with a mean age of 33 years (SD = 7.3 years). Drivers completed two drives, one each with the basic HMI without additional LED warning and a second with additional LED warning. The order of the two drives was permuted. This time the subjects experienced a TOR in two takeover situations (once with, once without traffic) in a separate practice drive before each of the two test drives to demonstrate the LED warning. This procedure was chosen since it could not be assumed that the warning was intuitively understandable. The parameters collected were identical to those in P2 except that subjects were asked about perceived situation criticality using the SCA scale [10] online after each takeover scenario.

**Fig. 9:**
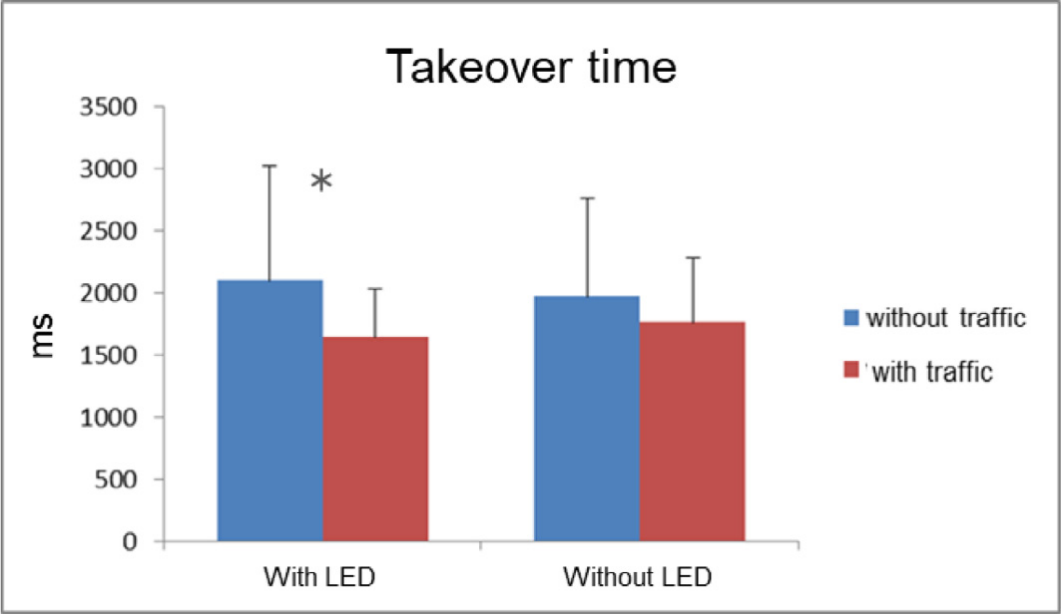
Takeover time dependent from the HMI variant (with additional LED in the exterior mirror or not) and the presence of approaching traffic on the neighbor lane (V1). * shows the significant effect of the factor “traffic” in the condition with LED.

In summary, V1 showed that the test setup is suitable for revealing specific HMI differences that aim to increase the driver's situation awareness: Faster takeover times were shown only in situations with traffic when an additional warning was given by means of an LED in the exterior mirror (see Fig. 9). The takeover usually consists of a braking maneuver, which is predominantly used as the method of choice for system deactivation, even if the situation itself did not require braking. A slightly lower tendency for unnecessary braking maneuvers was observed in scenarios without traffic when the LED warning was active. In those situations, it also seems to take somewhat longer until a first glance in a mirror is executed after the TOR. However, the takeover quality remained unaffected by the HMI condition due to the relatively large time budget for takeover. The HMI variation also had no effect on the subjectively perceived situation criticality.

#### Validation study 2 (V2)

The aim of validation study 2 was to investigate the effects of a reduction in the time budget for the takeover on the results (as V1 had shown differences in quantitative measures of takeover performance but no effects on qualitative measures). Therefore, the time budget from the moment of TOR to the collision with the obstacle was reduced from 8 s to 5.5 s. This time budget was selected based on the criterion to be noticeably different from the previous study but still controllable by driver intervention (which could be proven in some pretests). Otherwise, the test setup was similar to that in V1 to allow a comparison of the results. In order to generate a comparable criticality of the situations, the speeds of the rear traffic were adapted to the reduced time window.

The HMI variation investigated was the same as in V1. Again, two drives with vs. without additional LED warning were performed. Twenty subjects participated in the study (*n* = 10 female). The mean age of the sample was 34.6 years (SD = 8.3 years). The HMI was varied as a within-factor.

**Fig. 10:**
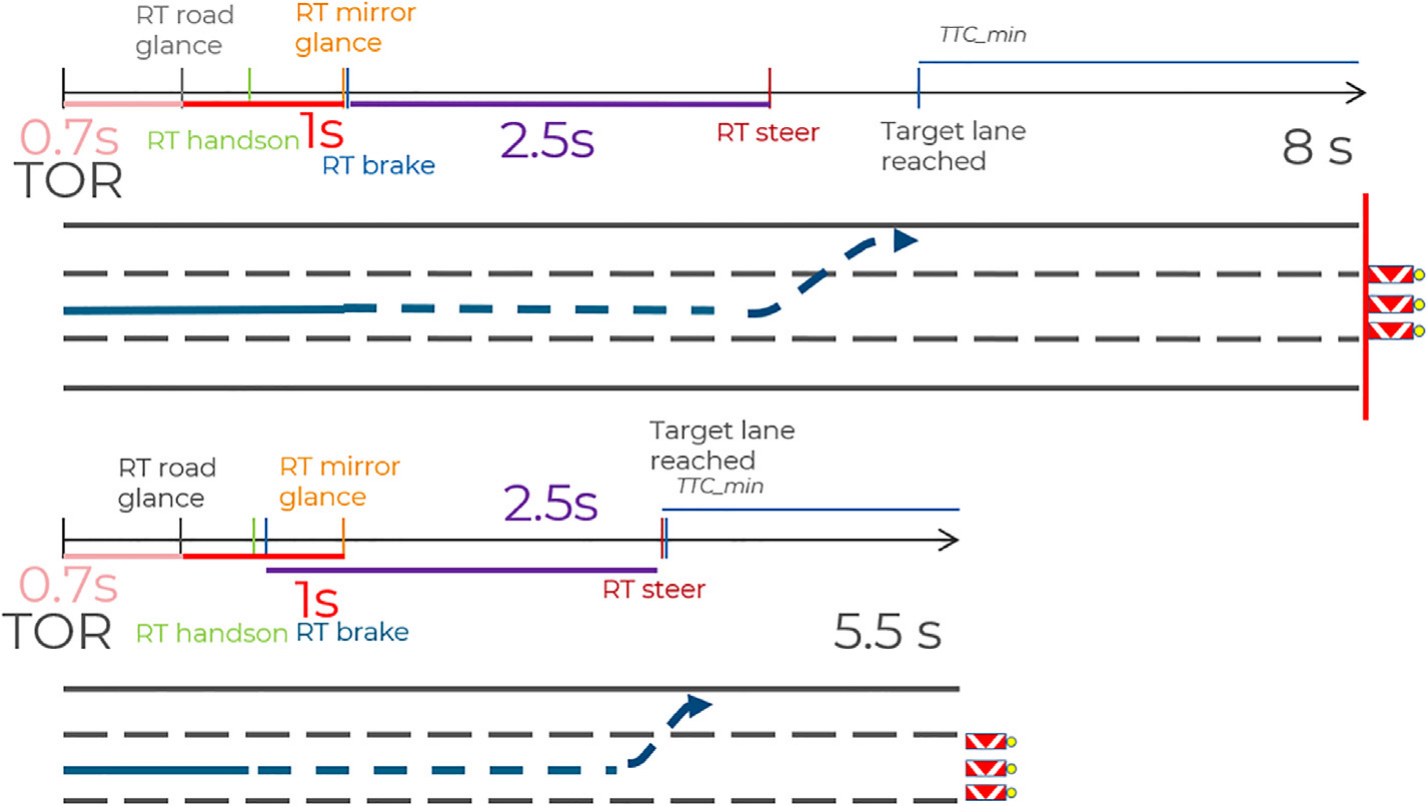
Schematic illustration of the comparison of times courses in mean reaction times between 8 s time budget in V1 vs. 5.5 s time budget in V2.

In general, the reduction of the time budget from 8 s to 5.5 s led to the following effects (results from V1 were compared with results from V2): Subjects tended to show an even stronger tendency to brake as initial reaction. This indicates that reducing the time budget made the reaction pattern even more homogeneous. Moreover, subjects showed faster and stronger takeover reaction with more critical outcome. Subjectively perceived criticality also increased. Furthermore, a stronger accumulation of critical situations occurred (especially collisions with the obstacle). The reaction time until the first glance in the mirror was comparable to that in V1. A more in-depth analysis of the mean reaction times (first glance at the road, hands-on-time, first glance in the mirror, initiation of braking and initiation of the steering maneuver) over the two compared time budgets yielded the result that, with a reduced time budget, the driver's reaction shifts forward in time to a point before the situation can be fully perceived and assessed (see Fig. 10).

Furthermore, the reduction of the time budget from 8 s to 5.5 s had the following effects on the sensitivity of the detection of HMI differences: The positive effect of the LED warning in terms of response times to the TOR disappeared (see, while positive effects on collision frequency with rear traffic remained. A subjectively slightly lower perceived criticality due to the LED warning also remained.

Overall, the results can be interpreted methodologically in such a way that a smaller time budget makes the takeover situation more critical overall, forcing drivers to react faster and stronger. However, the intended goal of the test setup, to map action decisions based on situation awareness, rather got lost because the drivers have to react so quickly that they may not have time enough to build up situation awareness. Hence, for the final definition of PADA-AD, the time budget was set to 8 s for L3 research questions.

#### Validation study 3 (V3)

The research question of validation study 3 was whether the test setup is also suitable to investigate Level 2 systems and corresponding human factor issues. One big difference of partially automated driving compared to L3 driving is that the drivers might also be confronted with situations where the system continues working towards an object/event undetected due to the functional limitation or the system suddenly terminates without warning the drivers. They have to detect these situations for themselves, and decide that they must take-over the driving task immediately from the system in such situations (driver-initiated take-over) by an adequate intervention in order to prevent a safety-critical outcome. Due to these technical constraints, the drivers must remain responsible for monitoring the traffic environment and are required to stay more involved in the driving task and to repeatedly monitor the driving environment compared to L3 driving. Typically, L2 systems therefore include a kind of Driver Monitoring System (DMS) that supervises the drivers’ monitoring behavior (either hands-on behavior only and/or visual attention behavior, i.e. whether the driver remains attentive to the forward road or looks away from the driving task for too long or for too often) and, if necessary, issues a warning if the driver fails to comply.

In order to check whether the test-up is suitable for also evaluating L2 systems, we changed the setup in a way that the required driver-initiated take-overs are addressed by presenting the scenarios without an immediate take-over prompt from the system. The remaining setup remained as described in the previous chapters. Using the setup, the following exemplary research question specifically for L2 systems was investigated: Can the method map the effects of different driver-in-the-loop strategies (DIL strategies) in L2? Driver-in-the-loop strategies refer to concepts that try to either keep the driver permanently in the loop while driving with active L2 (e.g., by means of a DMS) or to bring them back into the loop only if the situation deems it necessary (for example, by means of an indication that an unclear traffic situation is imminent). This issue is examined in a worst-case scenario in which the drivers are not attentive - although they always should be. Driver distraction was deliberately induced in the study by means of instruction.

In this study, drivers completed the test drive while using an L2 system. They were instructed to leave their hands at the wheel. During active L2, drivers watched a video without dialogues, but with an acoustic background. It was presented on a tablet mounted at glove compartment level. Both, gaze and head direction towards the display were used as indicators for distraction. To encourage subjects to focus their attention on the video, even though this is not allowed in L2 driving, they were explicitly instructed to do this for scientific purposes.

The study examined two different driver-in-the-loop strategies (DIL strategies) and compared them to a condition without any system intervention. This factor was implemented as a between factor.

**Fig. 11:**
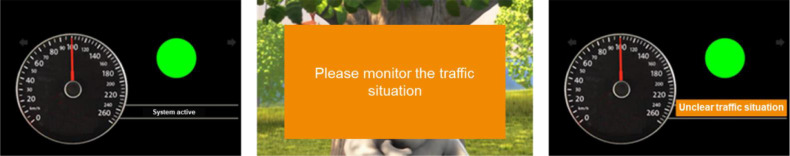
HMI variants in the different Driver-in-the-Loop strategies: Left: Baseline condition; Middle: eyes-off warning in the DMS condition; Right: Monitoring Request in the MR condition.
• Situation-dependent strategy (MR condition): The driver received a so-called “Monitoring Request” about an unclear traffic situation, which might require a possible intervention. It was given 10 s before the obstacle was reached (coinciding with the appearance of the situation). The message was displayed below the system status for 2 s and was combined with an audible advisory tone (see Fig. 11 right).
• No warning (Baseline condition): The driver received no warning or indication.

• State-dependent strategy (DMS condition): The drivers’ gaze behavior was monitored via DMS. If they look away from the road for too long (> 4 s), they receive an eyes-off warning. This message was displayed directly on the video screen as long as the driver looked to it. It consisted of a text box with the information “Please monitor the traffic situation” (see Fig. 11 middle).

As in the original setup, the obstacle and the rear traffic only appeared at a defined time point (here: 10 s before reaching the obstacle). The sample consisted of 30 subjects (13 female. Mean age: 41.5 years: SD = 13.2 years). *N* = 10 drivers each experienced one of the DIL strategies. After instructions about the L2 system, the NDRT and a short drive for practicing system deactivation subjects performed the test drive in their assigned test condition. All drivers were told to direct their attention to the video, but always on the premise that they must remain ready to intervene at all time.

The results revealed visible differences in the reaction times to the appearance of the situation (shorter gaze reaction times until perception of the situation and shorter intervention times) depending on the DIL strategy (with vs. without intervention). However, these effects were overlaid by strong individual differences in monitoring behavior during automated driving between drivers, so that none of these differences reached statistical significance. The qualitative analysis of single critical events indicated that in the condition without intervention, critical situations with the obstacle occurred more frequently or, conversely, such situations could apparently be successfully avoided by the interventions. In addition, differences in driver-in-the-loop strategies were also evident in drivers' subjective evaluations, e.g., that MR was perceived as more helpful in preparing for the upcoming situation.

The results further show that driver's performance of control glances during the interaction with NDRT is a highly individual and automated process which seems very difficult to be influenced by experimentally induced instructions and variations. In general, the experimental induction of distraction (in contradiction to legal aspects and driver's natural behavior) in order to investigate the effectiveness of possible countermeasures must be discussed.

## Conclusion

The following final conclusions on the developed test method PADA-AD can be derived:

• The method is suitable for evaluating HMI/driving state-related issues in the context of automated driving in the simulator.
• The test course enables the recording of the driver's takeover performance including higher cognitive processes.
• The PADA-AD Test maps the relevant steps of the takeover process by the following approaches.
• Perception: The chosen pop-up approach in the driving simulator environment (the obstacle and the rear traffic suddenly appear at the scene) maps the probability in L3 driving that drivers are fully out-of-the -loop during the automated drive and regain awareness not before the takeover request is perceived.
• Awareness: The test setup and the chosen takeover situations map the requirements on the driver to build up a proper situational model in every situation anew before the action is shown.
• Decision: The variation in the takeover scenarios across the test course requires the driver to take complex decisions on the appropriate actions: whether to change lanes at all, in which direction to change lanes, the number of required lane changes and the necessity to brake before the lane change.
• Action: The recommended measures map the complete takeover performance with regard to takeover speed, quality and subjectively perceived criticality.
• The method was primarily developed to be used for evaluating Level 3 systems (conditional automated driving) but can also be used for answering research questions regarding Level 2 systems (partially automated driving).
• The method primarily allows statements about relative comparisons between HMIs based on mean differences, but can also be used for absolute statements (e.g., based on the occurrence of worst-case events such as collisions).
• The method is efficient: the takeover performance is assessed within a 15 min test course.
• The method is highly controlled and thus replicable through reduced environmental conditions and "popping up” of situational elements.
• The method is (largely) standardized: predetermined test course with a fixed sequence of clearly defined situations, clear specifications for instruction, detailed specifications for the implementation of traffic.
• Certain aspects can be adapted to the specific issues as needed.
• The number/frequency of test scenarios within the course (e.g. for driver state-related questions).
• The type of non-driving related task during the automated drive.
• The amount of practice before the test runs.

The presented test setup is currently implemented in the driving simulation software SILAB 7.0. However, it should be easily adapted to other simulator software systems. For more detailed information on the method and the results of the performed validation studies see [1].

### Additional information: derivation of the test setup based on other approaches on standardizing takeover measurements

So far, there are only few approaches for standardizing takeover measurements. An approach for a standardized test setup for the assessment of (cognitive) takeover ability was described in [26]). The method was developed in cooperation with WIVW and successfully used in a simulator study at VW. The takeover situation is preceded by a phase of highly automated driving at 100 km/h on a three-lane highway in the simulation. During the automated drive, the projection of the driving scene is switched off so that the driver can only see a black screen. This is intended to create a standardized condition for the feeling of being "out of the loop." During automated driving, various non-driving related activities (NDRT) can be offered for the driver to complete. At the moment of the takeover request, the projection is then switched on again and the driver is confronted with the takeover situation. This consists of an obstacle in the middle lane ("moving construction site"), which is to be avoided by a lane change either to the left or to the right.

The complexity of the takeover situation can be varied by the design of the surrounding traffic. The relative speed and position of vehicles in the other lanes at the moment of the TOR were used to influence the decision which lane can be used safely without colliding with traffic in the neighboring lanes or with the obstacle. The instruction for the drivers was to take that direction for which they are not required to brake. One takeover per minute was issued, and a total of 18 takeovers were experienced per trip. Each HMI variation was examined in a separate drive. Gaze reaction times, hands-on times, takeover times, and takeover quality are recorded as dependent measures. A particular measure described in [26] is "cognitive" takeover time, which should express the time drivers need to cognitively process the situation until they are able to make the decision for a particular behavior. This was operationalized in the study via the verbalization of the direction the driver will choose before actually executing this lane change maneuver (the driver should say "right" or "left"). Applying this method, it was shown that both situation complexity and engagement in a NDRT had an impact on cognitive takeover ability.

A different test setup was developed by Wintersberger et al. [27]: They adapted the Lane Change Task from [28] which is typically used to measure the distraction effect of infotainment systems to the requirements of automated driving. In the original Lane Change Task, drivers change lanes every 150 m after the destination lane is announced by signs on the side of the road (thus a total of 20 times on a 3000 m stretch at a speed of 60 km/h). In the adapted version, subjects drive at 120 km/h on a three-lane highway while processing various NDRTs (chatting, watching a video). Instead of signs, the lane to be selected is shown in the head-up display at the moment of the TOR. The TOR was issued without any reason apparent to the subjects. The takeover time was 12 s, within drivers were expected to complete the lane change. Fourteen takeovers were experienced per trip, and the frequency of TOR was 1 min. Hand-on times, time to first driver response, time to change lanes, and number of omitted lane changes were used to evaluate takeover performance.

Gold used a kind of standard scenario to investigate takeover performance in a series of studies (Gold et al. [29]; Gold et al. [30]). During automated driving at 120 km/h, the driver processes an NDRT (the SuRT [8]). A vehicle in front obscures the driver's view of the situation while the vehicle is moving. At the moment of the TOR, this vehicle changes lanes and reveals the view of the situation. Only then does it become clear to the driver how or whether they must react. The scenario has been used to investigate different issues. In [29] for example, the effectiveness of a so-called monitoring request (a cue that prompts the driver to monitor the driving scene from which it is not clear whether it requires taking control of the vehicle) was investigated. For this purpose, critical situations in which a takeover was required were generated by pedestrians and obstacles. In another study ([30]), the scenario was used to investigate different takeover time budgets. Here, the situation always required a takeover, and the time budget for the takeover was either 5 or 7 s. Measures used to evaluate takeover performance were time to first driver response, mirror and road glances, time to actuation of the direction indicator, and type of response shown (steering, braking, both).

The PADA-AD Test presented here represents, in a sense, a mixture of the different approaches. In terms of the type of takeover scenario, a similar approach to [26] is chosen, in that the drivers must repeatedly take over the driving task in response to an obstacle in their own lane, and may have to consider rear traffic. This choice of takeover scenario also proves to be well suited after reviewing other literature sources. For example, in a literature analysis of the internal FAT literature database on automated driving [30], this scenario was identified as the most frequently used (of the *n* = 298 publications that provided information on the takeover scenario, 150 used an obstacle in the lane to be driven on). In addition, a classification scheme in the KoHAF project [32] defined this scenario as suitable for generating high urgency, low predictability, high criticality, and medium driver response requirements, as was desired here.

Also, with respect to the design of the automated drive, a similar approach to [26] is taken: The driver should not be able to anticipate the takeover scenario unless the TOR is issued. Instead of occluding the scenery, however, the out-of-the-loop feeling is created by engaging in a non-driving related activity. A similar approach was also taken by [33,34], or [35]. In addition, to prevent drivers executing control glances during the automated drive from recognizing the takeover situation in advance, the situation elements belonging to the takeover situation were only displayed in the scenery at the moment of the takeover request (pop-up approach). A similar approach was also chosen by Gold [28] in his test setup. This procedure is also intended to prevent the switching on of the projection itself from acting as a cue. Fog could have been used in a similar way, an approach as followed by [33,34]. The parameters evaluated, for example the type of takeover reaction (steering or braking), are also similar to those in [29,30] as is SuRT as the NDRT employed.

From the approach of [27] or the original method of [28] they are referring, the principle of a continuous test course was adopted, in which the driver has to execute lane changes repeatedly. However, Mattes' setup [28] aimed to evaluate the distraction effect from IVIS during manual driving, which is why the evaluations are mainly focused on parameters of continuous lateral guidance performance (degree of deviation from an ideal trajectory during lane changes), whereas the setup described here focuses on the discrete reaction speed and quality of lane changes in response to the TOR at the obstacle. In contrast to the application in [26], which does not use a real takeover scenario but only requires that the lane change is completed within a certain time budget, the plausible takeover scenario used here allows us to evaluate the action success in response to the TOR as a criterion for takeover performance.

A very rich source for recommendations on the design of experiments to investigate transition processes in the context of automated driving is the ISO Document TR21959-2 "Human Performance and State in the Context of Automated Driving: Part 2 - Considerations in designing experiments to investigate transition processes” [36]. It contains recommendations for the specifications of test scenarios (e.g. description of a scene by physical dimensions as well as the evolution of scenes in terms of a scenario, including the conditions preceding the actual test scenario to induce a certain driver state). We also used this approach for our description of the test scenarios. Also, the categorization of the test scenario into the most relevant psychological dimensions according to [31] KOHAF (urgency, predictability, criticality, complexity of the driver's required response) is referenced in the ISO document which we also considered in the selection of our test scenario. The ISO further includes a taxonomy of human performance measures which categorizes the measures according to the associated phases in the transition process, the addressed transition type (driver-initiated vs. system-initiated transfer of control) as well as the scope of assessment and data type considerations. In our approach we focused on the assessment of system-initiated transfer of control transitions on all assumed stages of the take-over process and included measures for the human response as well as vehicle response measures and their effects on traffic safety. The measures proposed in Table 2 are differentiated according to the addressed aspect of human performance (temporal measures vs. quality measures) and the source of the measures (subjective reports from the driver, measuring devices such as the driving simulator or an eye-tracking system).

## Declaration of Competing Interest

The authors declare that they have no known competing financial interests or personal relationships that could have appeared to influence the work reported in this paper.
